# Specificity of action selection modulates the perceived temporal order of action and sensory events

**DOI:** 10.1007/s00221-018-5292-5

**Published:** 2018-05-19

**Authors:** Andrea Desantis, Patrick Haggard, Yuji Ikegaya, Nobuhiro Hagura

**Affiliations:** 1 Département Traitement de l’Information et Systèmes, ONERA, Salon-de-Provence, France; 2 Laboratoire Psychologie de la Perception, CNRS, Université Paris Descartes, Paris, France; 3 Institute of Cognitive Neuroscience, University College London, London, UK; 4 Center for Information and Neural Networks, National Institute for Information and Communications Technology, Osaka, Japan; 5 Graduate School of Pharmaceutical Sciences, University of Tokyo, Tokyo, Japan; 6 Graduate School of Frontier Biosciences, Osaka University, Osaka, Japan

**Keywords:** Action awareness, Action selection, Time perception

## Abstract

The perceived temporal order of actions and changes in the environment is crucial for our inferences of causality. Sensory events presented shortly after an action are more likely considered as self-generated compared to the same events occurring before action execution. However, the estimation of when an action or a sensory change occurred is a challenge for the human brain. This estimation is formed from available sensory information combined with internal representations. Researchers suggested that internal signals associated with action preparation drive our awareness of initiating an action. This study aimed to directly investigate this hypothesis. Participants performed a speeded action (left or right key-press) in response to a go-signal (left or right arrow). A flash was presented at different time points around the time of the action, and participants judged whether it was simultaneous with the action or not. To investigate the role of action preparation in time perception, we compared trials where a cue indicated which action to perform in response to a later go signal presentation, and trials with a neutral cue where participants did not know until the time of the go signal which action to perform. We observed that a flash presented before the action was reported as simultaneous with the action more frequently when actions were cued than when they were uncued. This difference was not observed when the action was replaced by a tactile stimulation. These results indicate that precued actions are experienced earlier in time compared to unprepared actions. Further, this difference is not due to mere non-motor expectation of an event. The experience of initiating an action is driven by action preparation process: when we know what to do, actions are perceived ahead of time.

**Electronic supplementary material:**

The online version of this article (10.1007/s00221-018-5292-5) contains supplementary material, which is available to authorized users.

## Introduction

Causal inferences about an action and a consequence in the environment strongly rely on the perceived temporal order of these events. In fact, whether a sensory event occurs before or after an action can modulate causal perception, since causes must precede effects (Hume 1888; Wegner and Wheatley 1999). However, the estimation of when an action or a change in the environment occurred can be a challenge for the human brain. Since people do not have a sense dedicated to time, these estimations are formed from available sensory information combined with internal representations (Grondin 2010).

In line with this notion, researchers suggested that the estimation of when an action is initiated relies not only on re-afferent information but also on efferent signals associated with the action. For instance, when participants are asked to report the time at which they initiated a movement, they often report their action as occurring earlier in time compared to its actual onset time (Libet et al. 1983), or compared to a passive movement (Obhi et al. 2009; Strother and Obhi 2009). Action control models have often been used to explain these results. According to these models, the execution of an action involves the neural prediction of the expected sensory outcomes of the action (Wolpert 1997). The awareness of initiating an action would depend on the predicted sensory consequences of the movement, which are available before the actual sensory reafference (Blakemore et al. 2002). According to recent studies motor preparatory processes are considered to be responsible for the prediction of the sensory consequences of an action (e.g., Desantis et al. 2014; Ziessler and Nattkemper 2011). This suggests that the awareness of initiating an action may rely on action preparation processes.

Indirect evidence supporting the link between awareness of action initiation and action preparation comes from a study showing that people’s reports of when they executed an action tended to covary with lateralized readiness potentials (LRP), which are thought to reflect lateralized action-specific activation in the primary motor cortex (Coles 1989; Haggard and Eimer 1999; Leuthold and Jentzsch 2002): the earlier the mean LRP, the earlier participants’ awareness of having initiated an action (Haggard and Eimer 1999). Accordingly, specific action preparation might be involved in the experience of initiating a movement. In line with this notion further studies suggested that people’s awareness of initiating an action depends on the level of preparation their action reached (Ganos et al. 2015): the more a specific action is prepared, the higher the expectation that the action is going to be performed, the earlier the experience of initiating it. The present study aimed at directly investigating this hypothesis by assessing whether the preparation of a specific action leads participants to experience their action earlier in time compared to non-specific action preparation.

The vast majority of studies investigating the influence of efferent signals on the experience of initiating an action often compared active and passive conditions (see above). However, Hughes and his colleagues showed that active and passive movements do not only differ in terms of action processes, but also in terms of other processes such as attention and non-motor prediction (Hughes et al. 2013). Accordingly, in the present experiment in order to isolate the role of action preparation on time perception, participants always performed an action, and we manipulated the degree to which a specific action could be prepared.

Then, to examine whether the preparation of a specific action leads to experiencing an action as occurring earlier in time, we combined a temporal simultaneous judgement task with a choice reaction time task. Participants responded to a left or a right arrow (go-signal) by executing a left or a right key-press, respectively. In cued trials, an arrow pointing in the same direction as the go-signal was presented before the go-signal, allowing participants to prepare the subsequent action. In uncued trials, however, a neutral cue was presented conveying no information regarding the action to perform at go-signal onset. Thus, participants could only prepare a specific action after the go-signal. During this task, participants judged whether a flash, presented before or after the action (key-press), was simultaneous with their action or not. If the awareness of initiating an action depends on the level of motor preparation a specific action has reached, participants would experience cued actions as occurring more frequently before the flash compared to uncued actions, since specific motor preparation for cued actions would be achieved earlier in time compared to uncued actions. Moreover, we tested the same task but replaced participants’ action with a tactile stimulation in order to exclude the effect of non-motor expectations on participants’ time judgments.

## Materials and methods

### Participants

Twenty-four participants volunteered for the study (21 males; average age 27.83, SD 5.42) for a payment of 3000 JPY. All had normal or corrected-to-normal vision and were naïve as to the hypothesis under investigation. They all gave a written informed consent. Three additional volunteers participated in the study but they were not included in the sample and analyzed (see “Data analyses” for inclusion criteria). All experimental procedures were approved by the ethics committee of the National Institute of Information and Communications Technology (NICT).

### Apparatus and stimuli

Participants were tested using a personal computer (Lenovo, T400). Stimulus presentation and data collection were performed using Matlab software (R2013b) and the Psychophysics Toolbox (Brainard 1997; Pelli 1997). Tactile stimulation was delivered to the palmar part of the distal phalanx of the right hand thumb and fifth digit (12 mm^2^ contact surface) by solenoid tappers (Heijo Research Electronics), which were controlled with Matlab. In order to cover the noise made by key-presses and tappers, participants were presented with a background white noise via headphones.

**Fig. 1:**
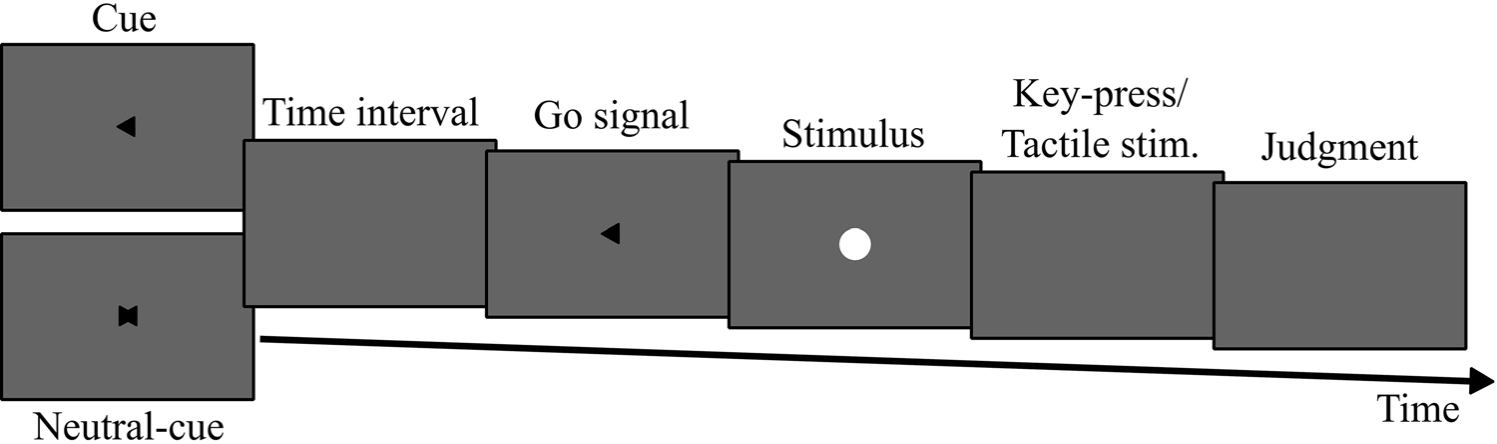
Illustration of an experimental trial. A trial started with a cue. In the cued trials, the cue pointed either left or right and indicated the action (left or right key-press) participants had to execute at the presentation of the go-signal (action condition), or the location of the tactile stimulation (thumb or 5th digit—tactile condition). In uncued trials, participants were presented with a neutral cue, i.e. the left and right arrows were superimposed (bottom left panel). Thus, participants did not know until the presentation of the go-signal-arrow what action to perform (action condition) or the location of the tactile stimulation (tactile condition). Before or after the action/tactile stimulation a white flash was presented (see “Procedure” for more details). Participants were asked to report whether it was presented simultaneously with the action/tactile stimulation or not

All visual stimuli were presented on a display with 1440 × 900 pixels resolution and 60 Hz refresh rate, from a viewing distance of approximately 55 cm. Visual flash stimuli consisted of white flash presented within a Gaussian envelop of about 1.5° diameter. Other visual stimuli consisted of two black arrows (with a shape of an equilateral triangle) pointing either to the left or the right, and a geometric shape created by the superimposition of the left and right arrows. The size of these stimuli was ~ 1.01° width (Fig. 1).

### Procedure

Participants completed two conditions: an action and a tactile condition. Each condition consisted of two blocks: cued and uncued blocks. Therefore, the experiment consisted of a 2 (ACTION: present, absent) by 2 (CUE: cued, uncued) factorial design. The order of the action and tactile condition, and the order of the cued and uncued blocks were counterbalanced across participants.

In each trial of the action condition, participants performed two tasks: two types of choice reaction time task (RT task) and a simultaneity judgment task (SJ task). In the RT task, participants were presented with an arrow at the center of the screen pointing either to the left or to the right (go-signal). The arrow was presented for two frames (33 ms). The orientation of the arrow was selected randomly and equiprobably. Participants were required to respond as fast and as accurately as possible to the arrow by executing a right-hand thumb key-press, when the arrow pointed to the right, and a right-hand fifth digit key-press, when the arrow pointed to the left. To manipulate the degree of action preparation in the RT task, two different types of cue-signal preceded the go-signal. In the cued block, before the presentation of the go-signal, participants were presented for 200 ms with an arrow pointing in the same direction as the go-signal (Fig. 1). The SOA between the cue-arrow and the go-signal-arrow was varied randomly and taken from an exponential distribution with average of 600 ms, and 500 and 800 ms for the shortest and longest possible SOAs, respectively. This was done to prevent any anticipation and temporally stereotyped response to the presentation of the go-signal. The uncued block was the same as the cued-action block except that participants were presented with a neutral cue (the result of the superimposition of the left and right arrows) before the onset of the go-signal (Fig. 1). Consequently, until the presentation of the go-signal, they did not know which action to execute. The trials in which participants pressed the wrong key were interrupted and repeated. Any key-press performed between 0 and 150 ms after the onset of the go-signal were considered as anticipation rather than reaction to the go-signal, which the trials were interrupted and repeated. Also, key-press performed later than the average reaction time + 480 ms was considered as a too late key-press and the trial was also interrupted and repeated.

For the SJ task, a visual flash (16 ms duration) was presented either before or after participants’ key-press executed in response to the go-signal. The flash was delivered randomly at one of ten different stimulus onset asynchronies (SOAs) ± 183 ± 150 ± 116 ± 83 ± 50 ms relative to the average action latency calculated on previous trials. Negative SOAs indicate that the flash was presented before the average reaction time. For the first trials, the average reaction time was calculated using the data from a short training session consisting of 20 trials, where participants were familiarized with the task. After the key-press was executed and the flash was presented, participants reported whether the flash occurred simultaneously with the action or not by pressing on one of two designated keys using their left hand. We chose simultaneity judgments, rather than temporal order judgements (“before” or “after” judgements) because the latter can lead to response biases that would affect directly our dependent measure (the point of subjective simultaneity, see “Data analyses”), such as tendencies to judge the flash more frequently as occurring after the action (see “Discussion”).

Crucially, the average reaction time, that was used to determine the onset of the flash, was calculated separately for each condition (cued and uncued) and finger (thumb and fifth digit key-press), in order to make sure that the distributions of action-flash SOA in respect to the reaction time were similar across conditions and the action executed.

The procedure for the tactile condition was the same as for the action condition, except that participants did not execute any key-press in response to the go-signal, but instead, were delivered with a tactile stimulation on the finger indicated by the arrow. At the end of the trial they judged whether the flash and the tactile stimulation were simultaneous or not. In order to maximally match the temporal parameters of the action and the tactile conditions, both the onset and the duration of the tactile stimulation were determined by the individual action onset and duration recorded in the action condition. For instance, the onset and duration of the thumb tactile stimulation in one of the trials of the cued-tactile block was determined by the onset and duration of a thumb key-press in one of the trials of the cued-action block. However, if a participant began the experiment with the tactile condition, the onset time and duration of the tactile stimulation was derived from the mean and standard deviation of all previous participants’ action latency and duration.

The experiment lasted about 90 min. Each condition (action, tactile) consisted of 600 trials: 30 trials per action/touch-flash SOA per Cue [30 (trials) × 10 (SOAs) × 2 (cues: cued and uncued)].

### Data analyses

Considering that visual flashes were presented on the basis of estimated action time (i.e., average action latency), we calculated the actual action-flash interval for each trial. Then, we divided each the stimulus-before-action trials and the stimulus-after-action trials into five time intervals of equal number of trials (total of 10 bins). This was done both for cued and uncued trials, for each participant (See Table 1 for mean and standard deviation of number of trials for each time bin, and mean and standard deviation of time intervals for each bin). The same procedure was performed for the tactile trials.

**Table 1:** Mean (SD) number of trials for each of the ten resampled bins in the action block and tactile block

Conditions	Bin 1	Bin 2	Bin 3	Bin 4	Bin 5	Bin 6	Bin 7	Bin 8	Bin 9	Bin 10
Number of trials										
Action block										
Cued	29.3 (1.3)	29.3 (1.3)	29.3 (1.3)	29.3 (1.3)	29.8 (2.1)	30.7 (1.3)	30.7 (1.3)	30.7 (1.3)	30.7 (1.3)	30.2 (2.1)
Uncued	28.6 (2)	28.6 (2)	28.6 (2)	28.6 (2)	28.7 (2.8)	31.4 (2)	31.4 (2)	31.4 (2)	31.4 (2)	31.3 (2.8)
Tactile block										
Cued	27.6 (1.9)	27.6 (1.9)	27.6 (1.9)	27.6 (1.9)	27.7 (2.5)	32.4 (1.9)	32.4 (1.9)	32.4 (1.9)	32.4 (1.9)	32.2 (2.5)
Uncued	29 (1.2)	29 (1.2)	29 (1.2)	29 (1.2)	28.9 (2)	31 (1.2)	31 (1.2)	31 (1.2)	31 (1.2)	31.1 (2)
SOAs (ms)										
Action block										
Cued	− 236 (39)	− 159 (16)	− 117 (13)	− 80 (12)	− 38 (7)	29 (9)	82 (11)	123 (14)	163 (18)	224 (27)
Uncued	− 231 (27)	− 155 (12)	− 114 (10)	− 77 (10)	− 36 (6)	26 (6)	81 (9)	122 (12)	163 (16)	223 (25)
Tactile block										
Cued	− 215 (34)	− 136 (27)	− 90 (31)	− 57 (25)	− 28 (12)	16 (16)	50 (31)	85 (40)	130 (36)	205 (28)
Uncued	− 229 (21)	− 155 (10)	− 114 (7)	− 74 (7)	− 33 (3)	26 (5)	82 (7)	123 (9)	163 (11)	222 (15)

Average (SD) SOA for each bin in the action block and tactile block

Then, we calculated the proportion of judgments action/touch and flash simultaneous for each time interval (10 time bins) per participant. From these data, psychometric functions were calculated using a (Gaussian) nonlinear regression model (Eq. 1) implementing the Maximum Likelihood procedure as described in Myung (2003). Three parameters were fitted (1) mean α, (2) standard deviation σ and (3) a scale factor *s*, which refers to the amplitude of the Gaussian curve.

1 $$f\left( x \right)=s \cdot {e^{ - {{\left( {x - \alpha } \right)}^2}/(2{\sigma ^2})}}$$

Importantly, to make sure that our binning procedure did not introduce any bias in the observed results we also analysed the data without binning the data at all (see Supplementary Material).

Each individual point of subjective simultaneity (PSS), representing an estimate of the temporal offset between the action/touch and flash required to perceive these two events simultaneously, was evaluated as mean of the fitted Gaussian curve. Standard deviation (SD) of the Gaussian curve was used as an estimate of participants’ temporal sensitivity, i.e. how well they could detect asynchronies between the action/touch and the flash. Higher SD values indicate low sensitivity to asynchrony. Participants were not included in the sample size if their PSS or SD (averaged across condition) was higher than the largest action/touch-flash SOA, since the fit of the Gaussian curve would be unreliable. Moreover, participants with a psychometric function whose amplitude was lower than 0.5 were rejected from further analyses due to the same reason. According to these criteria three volunteers participated in the study and were not included in the sample size.

Our main interest was the PSS difference between cued trials and uncued trials. We hypothesized that if action specification in cued trials leads participants to perceive their actions as occurring earlier in time, a significant change in PSS values would be observed in cued as compared to uncued-action trials. Moreover, we expected this effect to be driven by action preparation process, rather than the non-motor expectations; therefore, we expected no difference in PSS values between cued and uncued trials in the tactile condition. We tested this hypothesis by evaluating the interaction between the factors ACTION (present, absent) and CUE (cued, uncued).

After a first visual inspection of the PSS differences between cued and uncued trials for both action and tactile blocks we noticed the presence of an outlier. One participant had a PSS difference between cued and uncued trials exceeding the group average by 2.5 standard deviations. It is well known that mean values can strongly be affected by outliers; hence we decided to use non-parametric two-tailed exact Wilcoxon signed rank test for our analyses. This test is less affected by outliers since it relies on ranks rather than mean values. Significance value was set at *p* < 0.05 for all statistical tests. We also confirmed that excluding the outlier and performing a parametric test did not change the conclusion drawn from the non-parametric test.

## Results

Firstly, we verified whether our cues successfully facilitated action preparation. We compared mean reaction times (RTs) observed in the cued- and uncued-action trials. RTs were faster in the cued (*M* = 324 ms; SD 47 ms) than the uncued trials (*M* = 405 ms; SD 34 ms) (signed rank 3, *p* < 0.001). This shows that the manipulation of action preparation with the cues was successful.

**Fig. 2:**
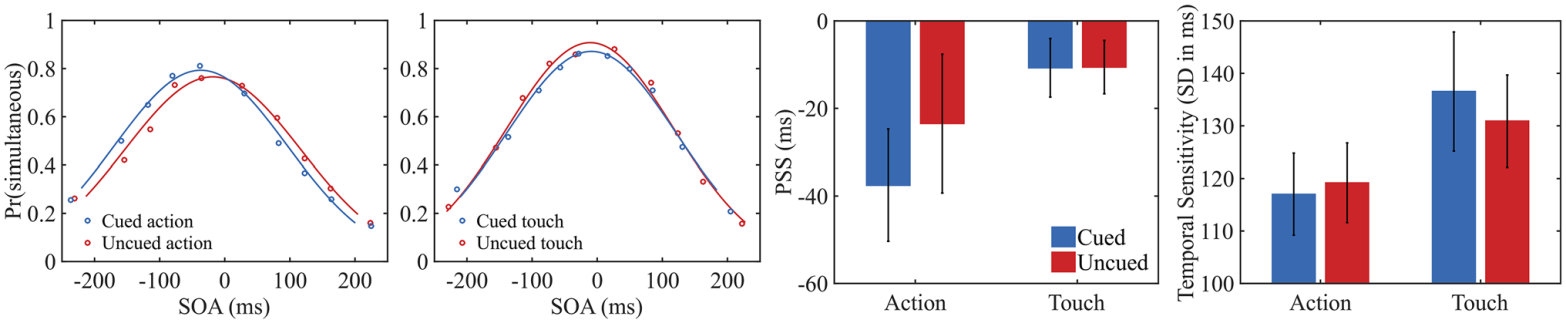
The first two graphs on the left depict psychometric functions for both the action and the tactile condition averaged across participants. The *y*-axes represent the proportion of judgments “action/touch and flash simultaneous” as a function of 10 time bins (*x*-axes) for both cued and uncued trials. Negative values indicate that the flash occurred before the action/tactile stimulation. Average r^2^, as estimates of goodness-of-fit, are reported as follow: cued-action 0.952; uncued-action 0.933; cued-touch 0.926; uncued-touch 0.917. The two graphs on the right depict average PSS and temporal sensitivity values (higher values indicate lower sensitivity) for each condition. Bars represent standard errors across participants

Our main interest, the ACTION (present, absent) × CUE (cued, uncued) interaction effect, was assessed performing a signed-rank test on the PSS difference between cued- and uncued-action trials compared to the same difference in the tactile trials (we report in the footnote^1^ the same analyses with parametric statistics performed on our sample without the outlier). The analyses showed a significant ACTION × CUE interaction (signed rank 71, *p* = 0.023). No main effect of ACTION (absent, present) nor CUE was observed (signed rank 102, *p* = 0.178, and signed rank 101, *p* = 0.169, respectively). Further analyses showed that the comparison between cued (*M* = − 37 ms, SD 63 ms) and uncued action trials (*M* = − 24 ms, SD 78 ms) was significant (signed rank 73, *p* = 0.027; Fig. 2). In contrast, in the tactile condition, no significant difference was observed between cued (*M* = − 11 ms, SD 33 ms) and uncued trials (*M* = − 10 ms, SD 30 ms), (signed rank 159, *p* = 0.811). These results show that a flash presented before the action was reported as simultaneous with the action more frequently when actions were prepared (cued actions) compared to when they were not prepared (uncued action).

Is one’s own action perceived ahead of the actual onset time of the button press, when the action is cued? To address this point, we conducted a one-sample signed rank test on the PSS values observed in each condition to evaluate whether cued actions were perceived earlier in time compared to their actual onset time (i.e., 0 ms). The analyses showed that PSS values observed in cued-action trials were significantly different from 0, signed rank 60, *p* = 0.010. None of the other tests reached significance (*p* > 0.1). This indicates that participants not only experienced their key-presses as occurring earlier in time in the cued compared to the uncued condition, but also that cued actions are experienced as occurring ahead of their physical time onset.

Bias in the mean of SOA distribution can influence the PSS of temporal order judgments (Miyazaki et al. 2006; Yamamoto et al. 2012). Given that RTs were faster in the cued compared to the uncued condition, it might be possible that the distribution of flash-action SOAs in respect to the reaction time differed between these two conditions. This difference might have led to the PSS shift we observed in the action condition. To test whether the distribution of action-flash SOA differed between cued- and uncued-action conditions we computed a two-sample Kolmogorov–Smirnov test. No difference between cued and uncued action-flash time distributions was observed (KS test 0.027, *p* = 0.999). The same analyses on touch-flash time distributions showed similar results (KS test 0.100, *p* = 0.093). Therefore, the PSS difference in the action condition cannot be attributed to differences in the SOA distributions. This also shows that our yoking procedure was successful (see “Procedure”).

Finally, we analyzed participants’ temporal sensitivity to flash-action/touch asynchrony (i.e. standard deviation of the Gaussian curve). We observed a main effect of ACTION (signed rank 249, *p* = 0.003). Participants were more sensitive to temporal asynchronies in the action condition (*M* = 118 ms, SD 35 ms) compared to the tactile condition (*M* = 134 ms, SD 46 ms). The main effect of CUE (cued, uncued) and the ACTION × CUE interaction were not significant. This shows that participants had higher temporal resolution when performing actions compared to when they passively received tactile stimuli.

The same results as those reported here were observed with psychometric functions directly fitted on simultaneity judgment gathered at each action-flash SOA (see Supplementary Material for analyses and figures).

## Discussion

Our study investigated whether preparing a specific action alters the temporal estimation of action initiation and in turn the perceived temporal order of action and a concomitant sensory event. We compared a condition in which participants could prepare a left or a right key-press (cued trials), with a condition in which this preparation was not possible (uncued trials). We observed that a flash presented before the action was reported as simultaneous with the action more frequently when actions were prepared (cued actions) compared to when they were not prepared (uncued action). This change in the perception of simultaneity was not observed when participants did not perform any action but instead were delivered with a tactile stimulation. Our results suggest that the early selection of a specific action can lead to the earlier perception of action initiation.

Note that the cue in the action condition did not only indicate which action to perform, it also provided information regarding the location of an upcoming event, i.e. the left/right action reafference. Previous studies showed that attended sensations are perceived better and faster than unattended sensations (Carrasco et al. 2000; Posner 1980). Therefore, the observed PSS difference in the action condition could be induced by a difference in the allocation of attention to the action reafference in cued compared to the uncued action trials. Notably, participants would process faster action reafferences in the cued compared to uncued action trials. In our study, however, the PSS difference was observed only in the action condition, but not in the tactile condition where the same cue informed about the location of the upcoming tactile stimulation. This dissociation suggests that the current result cannot be explained by attention towards the tactile reafference, but it is likely linked to action selection processes.

The PSS bias in the action condition could be induced by an overall bad estimation of simultaneity, due to the increased action related neural noise level (Harris and Wolpert 1998). However, this explanation seems unlikely, since sensitivity to temporal asynchronies (standard deviation of the psychometric function) did not differ between the cued- and uncued condition. Furthermore, sensitivity was overall higher in the action compared to the tactile condition, suggesting that participants had better temporal resolution when they performed actions compared to when they were delivered with a tactile stimulus. This corroborates the idea that action selection processes are responsible for the PSS change we observed.

Recent studies showed that action selection/preparation can influence participants’ experiences of agency (Chambon and Haggard 2012; Sidarus et al. 2013). In Chambon and Haggard (2012) participants reported high experiences of control over a visual stimulus, when action selection/preparation was facilitated by subliminal primes. Since a causal action must precede its effect (Desantis et al. 2016; Timm et al. 2014), the PSS difference in our action condition may reflect some sort of causal bias towards considering the flash as the consequence of an action, when this is prepared compared to when it is not.

However, this interpretation is unlikely for the following reasons. Firstly, in our study, participants judged whether the action and the flash were simultaneous or not, rather than performing a temporal order judgement in which they would indicate whether the flash occurred before or after the action. This kind of ‘causal’ bias should affect in particular PSS values obtained in temporal order rather than in simultaneity judgment tasks, since it would lead participants to judge the flash as occurring more often “after” their action rather than “before” (i.e. causes must precede effects). Instead, in the present study, simultaneity judgments were orthogonal with respect to causal judgments, thus preventing the influence of a ‘causal’ bias on participants’ responses.

Secondly, previous studies examining the perceived temporal relationship between an action and a sensory event opted for a biased action-stimulus SOA distribution, in which sensory events occurred more frequently after the action (Stetson et al. 2006). However, in our study, the distribution of the action-flash SOAs is centred on the timing of the action, resulting in the flash occurring equiprobably before and after the action. Furthermore, the SOA distributions did not differ between the conditions. Therefore, the statistical structure of the flash–action relationship is neutral, which may prevent participants to generally consider flashes as the consequences of their actions. Based on these arguments, we are confident in suggesting that the PSS difference observed in the action condition is driven by action selection processes and reflected participants’ experience of initiating an action earlier in time when the action was cued compared to when it was not.

Our results can be explained in terms of action control processes. According to a widely accepted theory of motor control the execution of an action involves the prediction of the sensory outcome of the action (Wolpert 1997). In line with this theory, the awareness of initiating an action would depend on the predicted sensory effects of the action, which are available before the actual sensory reafference (Blakemore et al. 2002). Past research showed that the prediction of the sensory outcome of an action is generated by motor preparatory processes (e.g., Desantis et al. 2014; Ziessler and Nattkemper 2011). This might indicate that the awareness of initiating an action also rely on motor preparatory processes. In agreement with this hypothesis, Ganos et al. (2015) suggested that people’s awareness of initiating an action depends on the level of preparation an action reached: the more an action is specified, the more it is predictable, and the earlier it is experienced in time. The present study corroborates this notion. Given that specific motor preparation for cued actions was achieved earlier in time compared to uncued actions, participants experienced cued actions as occurring more frequently before the flash compared to uncued actions. Accordingly, premotor and posterior parietal regions might be responsible for the effect we reported here. Indeed, it has been shown that the perceived timing of action is linked to the latency of lateralized readiness potentials (Haggard and Eimer 1999), which reflects the preparation/execution of specific actions in the premotor/primary motor areas (Coles 1989; Leuthold and Jentzsch 2002). Moreover, the perceived timing of action can be delayed by transcranial magnetic stimulation applied to the medial frontal regions (FCz), likely affecting high-order motor areas, such as supplementary motor areas (Haggard and Magno 1999). Finally, direct intracranial stimulation studies suggested that posterior parietal regions play a crucial role in the awareness of action (Desmurget and Sirigu 2009). Therefore, we speculate that a network of premotor and posterior parietal regions might underlie our effect.

A second possible explanation is that the observed effect is not driven by an increase of motor preparatory activity in premotor and parietal areas but rather to inhibitory prefrontal activity (Di Russo et al. 2017). Notably, it can be suggested that uncued actions are not less prepared than cued actions and that the longer reaction times for uncued actions are due to the inhibition of the key-press that participants are not required to perform until the final selection of the correct movement. This would be associated to inhibitory activity in the prefrontal cortex rather than to an increase of motor preparation in premotor areas (Di Russo et al. 2017). Further studies combining brain imaging and behavioral methods are required to define the role of motor preparation and action inhibition in the effect observed here. However, even though the specific mechanisms (preparation or inhibition) underlying the effect we observed remains unclear, the current results clearly indicate that the fluency of action selection (defined as the selection of the required action and the inhibition of the alternative) shapes our experience of initiating an action.

Our findings corroborate past research showing that the consciousness of initiating an action anticipates the actual onset time of the movement (Libet et al. 1983; McCloskey et al. 1983; Obhi et al. 2009), but they do not exclude the contribution of afferent signals to the perception of action timing. Past research pointed out that the awareness of initiating an action is not uniquely driven by motor preparatory/inhibitory activity but also to some extent by reafferent signals (Obhi et al. 2009). Therefore, it can be postulated that the experience of action initiation is realised by the weighted average of both efferent and afferent inputs via Bayes rule. Our study highlights the contribution of internal preparatory/inhibitory signal associated with action selection processes to our experience of action initiation.

In conclusion, our findings suggest that the fluency of action selection shapes the experience of initiating an action. Movements that were cued and selected in advance were perceived to occur earlier than those that were not. Any factor that causes actions to be perceived earlier, such as prior cue in in the present study, may also cause a stimulus presented before action to be erroneously perceived as occurring after action. Thus, while cueing and action selection clearly have benefits in terms of speed and accuracy of motor responses, they may also have a hidden cost, because they may lead to erroneous attribution of an external event to one’s own agency. Differences between trials, conditions, or individuals in selection of action might alter the perceived temporal order between actions and plausible sensory events, potentially leading to a misattribution of agency. We have not directly measures sense of agency in this study, so further studies should address the link between agency and the present effects of action selection on experience of action.

## Electronic supplementary material

Below is the link to the electronic supplementary material.

Supplementary material 1 (DOCX 113 KB)

## Footnotes

1 A repeated measure ANOVA with ACTION and CUE as factors showed a significant Interaction ACTION × CUE F(1,22) = 8.779, *p* = 0.007. Two-tailed paired-sample *t* test showed a significant difference between cued and uncued-action condition (*p* = 0.006). No difference was observed in the tactile trials (*p* = 0.808).
