# Supplementary material for: Specificity of action selection modulates the perceived temporal order of action and sensory events
Source: Exp Brain Res. 2018 May 19;236(8):2157–64. doi: 10.1007/s00221-018-5292-5 (PMC6061496; doi:10.1007/s00221-018-5292-5)
Supplement: Supplementary file 1 — Supplementary material 1 (DOCX 113 KB) [file 221_2018_5292_MOESM1_ESM.docx]

**Specificity of action selection modulates the perceived temporal order of action and sensory events**

Andrea Desantis ^1,2,3,4^, Patrick Haggard ^3^, Yuji Ikegaya ^4,5^ & Nobuhiro Hagura ^4,6^

^1^ Département Traitement de l’Information et Systèmes, ONERA, Salon-de-Provence, France

^2^ Laboratoire Psychologie de la Perception, CNRS, Université Paris Descartes, France

^3^ Institute of Cognitive Neuroscience, University College London, UK

^4^ Center for Information and Neural Networks, National Institute for Information and Communications Technology, Osaka, Japan

^5^ Graduate School of Pharmaceutical Sciences, University of Tokyo, Tokyo, Japan

^6^ Graduate School of Frontier Biosciences, Osaka University, Osaka, Japan

**Analyses & Results**

In the manuscript, PSS values were calculated by binning participants’ simultaneous judgment. To ensure that this binning procedure did not bias our results we calculated psychometric functions directly from each simultaneity judgment given at each time point, thus without binning the data at all. Psychometric functions were calculated using a (Gaussian) nonlinear regression model (eq. 1) implementing the Maximum Likelihood procedure as describe in Myung (2003). Three parameters were fitted 1) mean α, 2) standard deviation σ and 3) a scale factor *s,* which refers to the amplitude of the Gaussian curve.

(1) $f\left( x \right)={s\cdot e}^{-\left( x-\alpha\right)^{2}/(2\sigma^{2})}$

From each individual fit we estimated PSS and Temporal sensitivity. Our main interest, the ACTION (present, absent) x CUE (cued, uncued) interaction effect, was assessed performing a signed-rank test on the PSS difference between cued- and uncued-action trials compared to the same difference in the tactile trials (we report in the footnote^[[1]](#footnote-1)^ the same analyses with parametric statistics performed on our sample without the outlier). The analyses showed a significant ACTION x CUE interaction (signed rank = 77, p = 0.037). No main effect of ACTION (absent, present) nor CUE were observed (signed rank = 100, p = 0.153, and signed rank = 99, p = 0.145, respectively). Further analyses showed that the comparison between cued (M = -37 ms, SD = 62 ms) and uncued action trials (M = -23 ms, SD = 77 ms) was significant (signed rank = 76, p = 0.034). In contrast, in the tactile condition, no significant difference was observed between cued (M = -10 ms, SD = 33 ms) and uncued trials (M = -10 ms, SD = 31 ms), (signed rank = 154, p = 0.909). These results show that a flash presented before the action was reported as simultaneous with the action more frequently when actions were prepared (cued actions) compared to when they were not prepared (uncued action).

In addition, we conducted a one-sample signed rank test on the PSS values observed in each condition to evaluate whether cued actions were perceived earlier in time compared to their actual onset time (i.e., 0 ms) . The analyses showed that PSS values observed in cued-action trials were significantly different from 0, signed rank = 61, p = 0.011. None of the other tests reached significance (p > 0.1). This indicates that only when actions were cued participants experienced their keypresses as occurring earlier in time compared to their actual onset.

Finally, we analyzed participants’ temporal sensitivity to flash-action/touch asynchrony (i.e. standard deviation of the Gaussian curve). We observed a main effect of ACTION (signed rank = 61, p = 0.011). Participants were more sensitive to temporal asynchronies in the action condition (M = 117 ms, SD = 36 ms) compared to the tactile condition (M = 133 ms, SD = 49 ms). The main effect of CUE (cued, uncued) and the ACTION x CUE interaction were not significant. This shows that participants had higher temporal resolution when performing actions compared to when they passively received tactile stimuli.


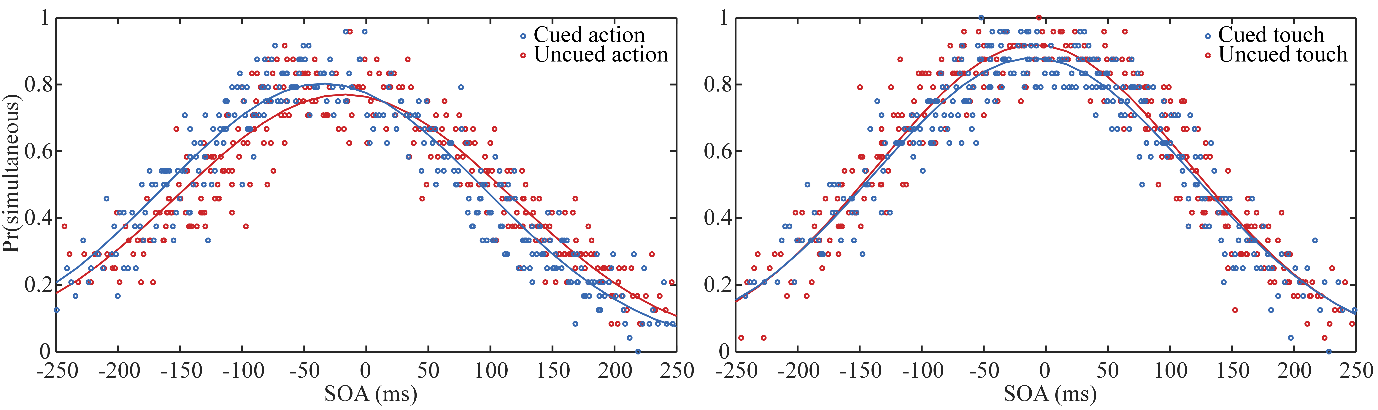


**Figure 1.** The graphs depict psychometric functions for both the action and the tactile condition pooled across participants. The y-axes represent the proportion of participants indicating that “action/touch and flash simultaneous” as a function of each action-flash SOA (x-axes) for both cued and uncued trials (300 SOAs per condition). Negative values indicate that the flash occurred before the action/tactile stimulation. Thus, each dots represent the proportion of participants (n/24) judging that the action and the flash were simultaneous at a given SOA, i.e. from the most negative to the most positive SOA.

1. A repeated measure ANOVA with ACTION and CUE as factors showed a significant Interaction ACTION x CUE F(1,22) = 7.986, p = 0.01. Two-tailed paired-sample t-test showed a significant difference between cued and uncued-action condition (p = .008). No difference was observed in the tactile trials (p = 0.776). [↑](#footnote-ref-1)
